# The K-turn motif in riboswitches and other RNA species^☆^

**DOI:** 10.1016/j.bbagrm.2014.04.020

**Published:** 2014-10

**Authors:** David M.J. Lilley

**Affiliations:** Cancer Research UK Nucleic Acid Structure Research Group, MSI/WTB Complex, The University of Dundee, Dow Street, Dundee DD1 5EH, UK

**Keywords:** Kink-turn, RNA structure, RNA folding, Tertiary interaction

## Abstract

The kink turn is a widespread structure motif that introduces a tight bend into the axis of duplex RNA. This generally functions to mediate tertiary interactions, and to serve as a specific protein binding site. K-turns or closely related structures are found in at least seven different riboswitch structures, where they function as key architectural elements that help generate the ligand binding pocket. This article is part of a Special Issue entitled: Riboswitches.

## Highlights

• The K-turn is a widespread structural motif in RNA, generating a tight helical kink.
• K-turns fall into two structural classes, depending on a critical H-bond acceptor.
• K-turns mediate tertiary interactions in RNA molecules.
• K-turns commonly bind specific proteins, exemplified by the L7Ae protein family.
• K-turns can be elaborated into more complex forms, including the k-junction.

## 1 Introduction

**Fig. 1:**
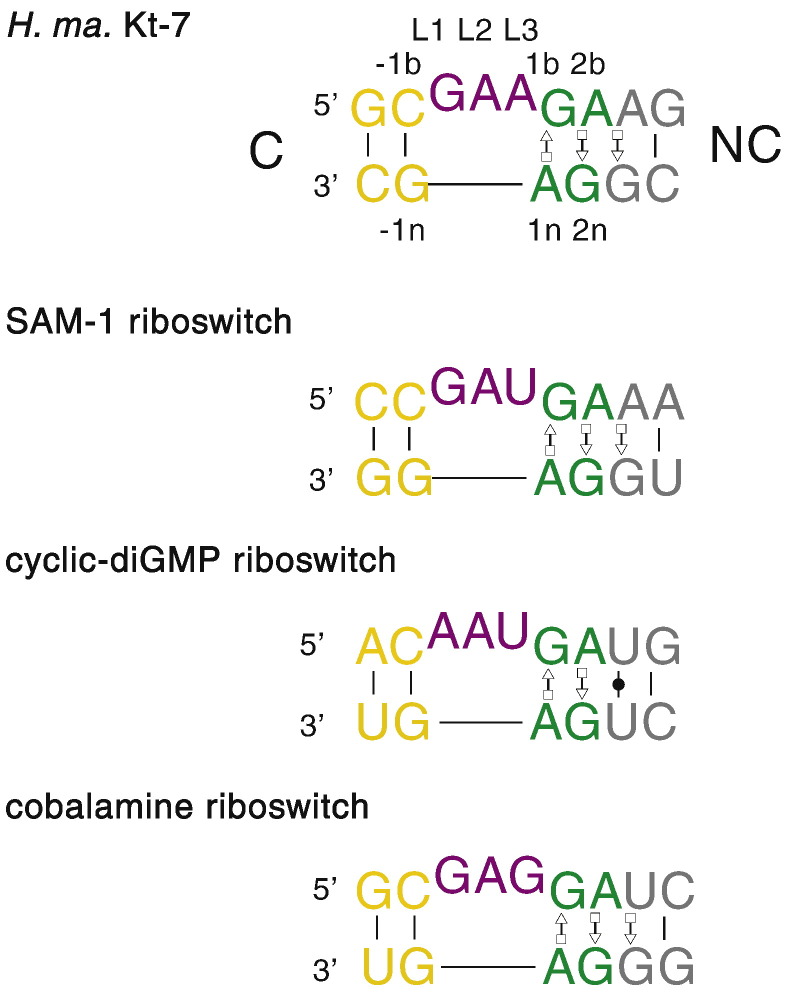
The sequence of standard K-turns. The sequence of *H. marismortui* Kt-7 is shown at the top, with the standard nomenclature for nucleotide positions [24]. The C and NC helices are indicated, the latter having the G·A pairs. The loop nucleotides are designated L*n* with *n* increasing 5′ to 3′. The remaining nucleotide positions are indicated by the suffix b for the bulge-containing strand, and n for the other strand, and positions 3′ to the bulge (i.e. the NC helix) are positive and 5′ to the bulge (C helix) are negative. The sequences of the SAM-I, cyclic-diGMP and cobalamine riboswitch K-turns are shown. Each is a standard, simple K-turn.

**Fig. 2:**
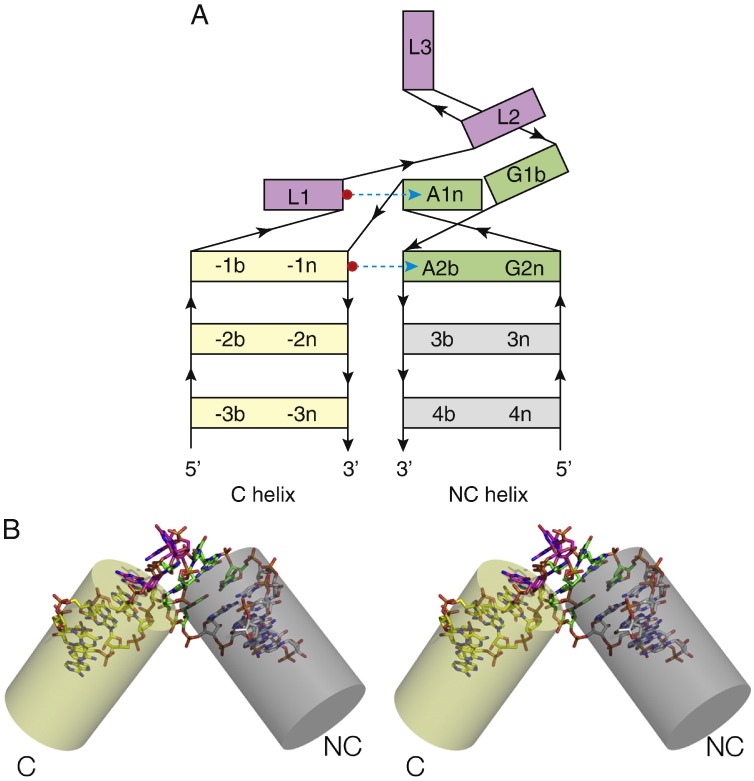
The global structure of a K-turn. A. Schematic to show the structure of a folded K-turn. The axis strongly kinks about the loop, with the adenine nucleobases of the G·A pairs directed towards the C helix. The L1 and L2 bases stack onto the ends of the C and NC helices respectively. The same color coding is used in all the molecular graphic images i.e. G·A pairs—green, NC helix—gray, C helix—yellow and loop—magenta. The broken arrows (cyan) indicate two key cross-strand hydrogen bonds donated by the O2′ of the L1 and − 1n nucleotides, discussed further below. B. Parallel-eye stereographic image of a folded K-turn structure, showing the strong axial kink. The kink angles for K-turns tightly cluster around 50°.

The kink turn (abbreviated to K-turn or k-turn, depending on the whim of reviewers) is a widespread structural motif in RNA, including many of the riboswitches. It is found in double-helical RNA, frequently consisting of a three-nucleotide bulge flanked on the 3′ side by a G·A pair, an A·G pair and often one or two further non-Watson–Crick pairs before regular basepairing resumes (Fig. 1). This helix is called the NC (non-canonical); the helix on the 5′ side of the bulge is called the C (canonical) helix. The K-turn generates a kink between the C and NC helical axes (Fig. 2), with an included angle of ~ 50°. K-turns had been described as a repeated sequence [1] or a kinked structure [2] in earlier studies, but the structure was first identified as a new motif when Steitz and colleagues found six examples in the 23S rRNA of the *Haloarcula marismortui* ribosome, and noted two cases in the 16S rRNA of *Thermus thermophilus* [3].

## 2 The occurrence of K-turns

K-turns are extremely common in functional RNA molecules. They have been found in a number of riboswitches (Fig. 1); SAM-I [4], cyclic-diGMP [5], [6], cobalamine [7] and T-box [8] riboswitches have known K-turn structures, while in some species lysine [9] and glycine [10] riboswitches have putative K-turn sequences, and some TPP riboswitches have an elaborated form we call a k-junction [11], [12], [13]. They are clearly a useful structural motif for building RNA structures that can bind small molecules with great specificity. However, K-turns are used much more widely, and are found in almost all kinds of functional folded RNA species including very large RNA-protein assemblies. In addition to the ribosomal examples [3], K-turns occur in the nucleolar RNAs that direct RNA modification [14], [15], [16], [17], the spliceosomal U4 snRNA [2], [18] and in untranslated mRNA regions [19], [20], including the riboswitches as noted above. Other functional RNA species contain known or predicted K-turn-forming sequences, including the human signal recognition particle [21] and ribonuclease P [22]. K-turns are thus implicated in almost every aspect of RNA function, including the modification and translation of RNA, the control of gene expression and the assembly of spliceosomes. We provide an interactive database of K-turn sequences and structures that is on-line at http://www.dundee.ac.uk/biocentre/nasg/kturn/ [23].

## 3 Classification of K-turns

**Fig. 3:**
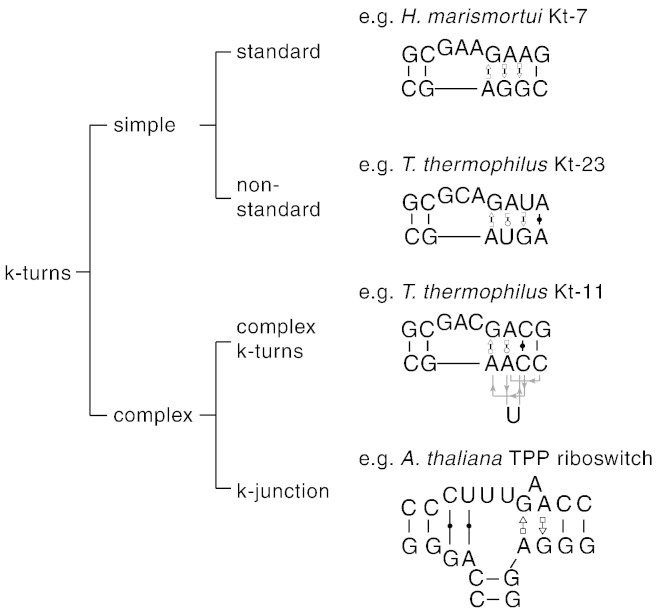
A classification of K-turn structures. K-turns can be divided into simple and complex. Examples are shown for each class. Simple K-turns are further divided into standard and non-standard, with the critical G·A pairs preserved or substituted respectively. The complex K-turns exhibit a greater departure from the standard K-turn, where the primary sequence does not map onto the 3D RNA structure. k-junctions can be considered as another branch of the complex K-turns. This scheme is taken from reference [13].

The K-turns can be classified into different groups based on sequence and structure (Fig. 3). The simple K-turn is a double-stranded RNA with a bulge that is followed by the A·G pairs of the NC helix. The nucleotides are named according to a universal scheme [24]. This is used throughout this review, and explained in Fig. 1. The simple K-turns may be subdivided into standard and non-standard classes. The standard simple K-turn has G·A and A·G pairs at the 1b·1n and 2b·2n positions respectively, exemplified by *H. marismortui* Kt-7 or the human U4 snRNA K-turn. Non-standard simple K-turns have a substitution in one of the G·A pairs. In Kt-23 of the ribosomal small subunit, the 2n position has a frequency of U > C > G > A over different species; yet where it was studied it has been found that they form normal K-turn structures despite the variation from the standard sequence [25], [26].

In some K-turns the nucleotides contributing to the G·A pairs do not map linearly onto the sequence of the RNA, although the structure formed is recognizably a normal K-turn. We term these complex K-turns. Applying our K-turn nomenclature [24], we assign nucleotides by their location in the 3D structure, as opposed to the primary sequence. In *T. thermophilus* Kt-11 the non-bulged strand of the NC helix doubles back on itself to form an S-turn, so that in the primary sequence the 1n and 2n nucleotides are separated by two nucleotides including the cytosine at the 3n position (Fig. 3). Nevertheless, the A2b is placed normally within the structure so that it accepts a hydrogen bond from − 1n O2′ to form an N1 class K-turn. In Kt-15 of *H. marismortui* the adenine that can be regarded as the 2b position (though its function is really somewhat different in this case) is actually contributed by the non-bulged strand, and a triple G2n·U·A2b interaction is formed. Yet the structure is still basically a K-turn, with a normal G1b·A1n basepair. We have recently identified a more extreme form of complex K-turn, where the non-bulged strand is interrupted by a third helix to form a three-way helical junction [13]. We therefore term this class of structures the k-junction.

It is interesting to note that a K-turn in a particular RNA within one organism may be functionally replaced by an alternative structure in a related species. For example, ribonuclease P RNA uses a tight kink in an RNA helix to make a loop–receptor interaction that creates the substrate binding site [27]. The ribozyme of some species uses a K-turn, while others have a structurally-unrelated element called the pk-turn. The pk-turn in *Thermotoga maritima* RNase P is globally a very similar structure to a K-turn, but lacks all the standard K-turn elements. Yet we have found that the pk-turn and K-turn can substitute functionally for one another i.e. a SAM-I riboswitch where the K-turn is substituted by the pk turn can bind SAM ligand, and *T. maritima* RNase P containing a K-turn in place the pk turn exhibits ribozyme activity [28]. Another example of such behavior is found in the lysine riboswitch. Lafontaine and coworkers [9] identified a putative K-turn-forming sequence in the aptamer domain of 32 lysine riboswitches, that once again facilitates tertiary interaction. They showed that the probable K-turn of *Bacillus subtilus* formed a bent structure as an isolated RNA duplex, and could be bound by the L7Ae protein. However, a K-turn is not present in the structure of the lysine riboswitch of *T. maritima* [29], [30]. Instead its structural role was fulfilled by an alternative kinked structure that lacks G·A pairs, lacking stabilization of the turn by long-range hydrogen bonds.

## 4 The structure of the simple K-turn

**Fig. 4:**
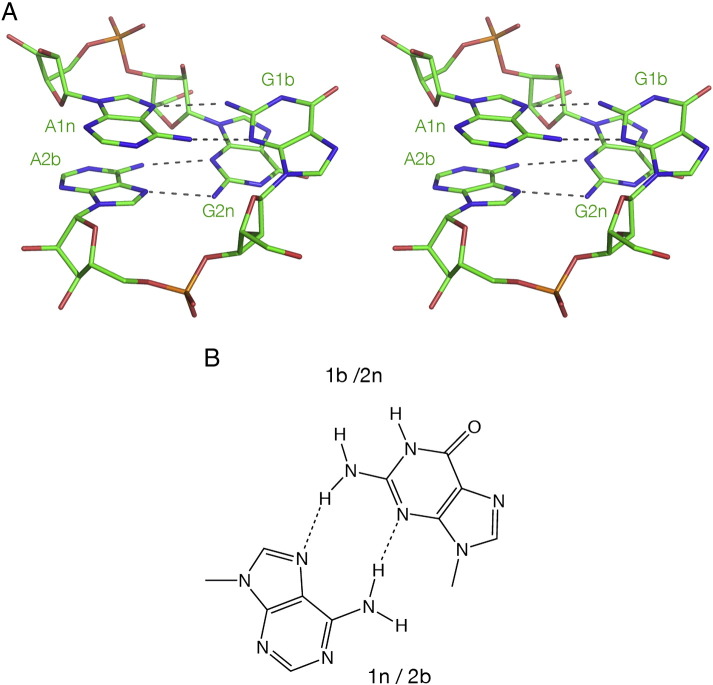
The G·A pairs of the K-turn. A. Parallel-eye stereographic image of the two G·A pairs of the box C/D K-turn [14], chosen as an example of an N3-type standard K-turn. B. Both G·A pairs are trans G(sugar edge)·A(Hoogsteen edge) pairs bonded by hydrogen bonds from GN2 to AN7 and AN6 to GN3.

The two G·A pairs at the 1b·1n and 2n·2b positions really form the core of the folded K-turn. Both are trans G(sugar edge)·A(Hoogsteen edge) pairs that are connected by hydrogen bonds from GN2 to AN7, and AN6 to GN3 (Fig. 4). However, it should be noted that the latter bond is not present in the 2n·2b pair in the N1 class of K-turns (see below). In general the 1b·1n is strongly buckled, with G1b turned ~ 25° out of the plane parallel to the other basepairs of the NC helix. By contrast the 2n·2b pair is approximately planar. Functional group substitution has shown that the hydrogen bonds of the G·A pairs are important to the stability of the folded K-turn. For example, guanine to inosine substitutions (obviating the formation of the GN2 to AN7 bond) at either position [31] prevents ion-induced folding.

In the folded K-turn, the 5′ nucleotide of the loop (L1) is stacked onto the end of the C helix, L2 is stacked onto the end of the NC helix, while L3 is directed away from the K-turn into the solvent (Fig. 3A). L2 adopts a syn conformation to maximize stacking on A1n.

**Fig. 5:**
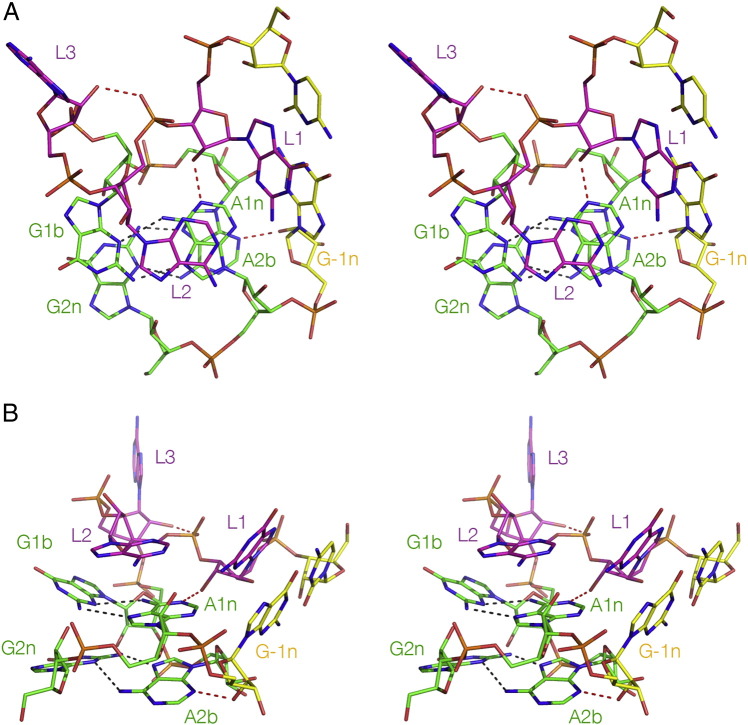
The cross-strand hydrogen bonds of a standard K-turn. Two parallel-eye stereographic views are presented for *H. marismortui* Kt-7 as a protein-free duplex species, which forms a standard N3-type K-turn structure [39]. Hydrogen bonds are indicated by broken lines. The two key cross-strand hydrogen bonds are donated by the O2′ of L1 and − 1n to A1n N1 and A2b N3 respectively. These, together with the bond from the L3 O2′ to the L1/L2 phosphate proS O are highlighted in red. A. View looking down onto the G·A pairs. B. View from the side of the non-bulged strand.

The folded structure is stabilized by a number of H-bonding interactions within the core [24], [31], [32], [33]. The minor-groove edges of the adenine nucleobases of the two A·G pairs are pointed towards the minor groove of the C helix to facilitate the formation of critical A-minor interactions [34], allowing the formation of two critical cross-strand hydrogen bonds (Fig. 5). One is donated from O2′ of L1 ribose to N1 of A1n in the G·A pair closest to the bulge. Removal of the O2′ atom (i.e. substitution by 2′-deoxyribose at that position) from the *H. marismortui* Kt-7 completely prevented Mg^2 +^ ion-induced folding [24]. The other is donated by the O2′ of the ribose in the − 1n position to a ring nitrogen atom of adenine at the 2b position. The acceptor for the hydrogen bond can be either N3 or N1, and this difference divides the known K-turns into two structural classes. This is discussed further in the following section.

**Fig. 6:**
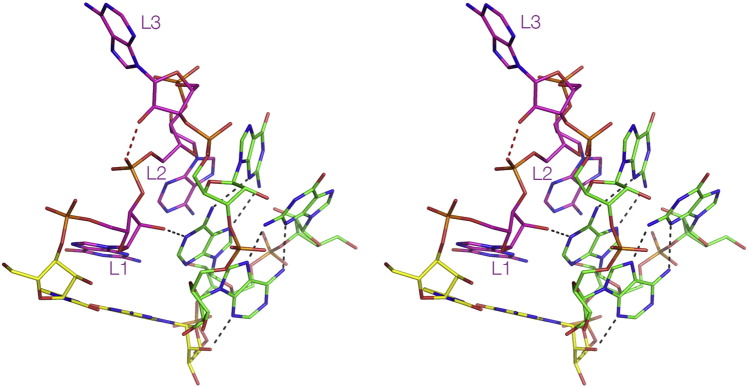
The loop *H. marismortui* Kt-7 as a protein-free duplex species [39], viewed from the side of the bulged strand as a parallel-eye stereographic image. The L3 O2′ to L1/L2 phosphate proS O hydrogen bond is shown by the broken line highlighted in red.

In many K-turns another hydrogen bond is observed, that closes the neck of the loop. The L3 O2′ donates its proton to the proS non-bridging O of the phosphate linking L1 and L2 (Fig. 6). Removal of L3 O2′ in *H. marismortui* Kt-7 resulted in an impairment of ion-induced folding [24]. Further hydrogen bonds can be found in various different K-turns. These appear to form adventitiously as the sequence permits.

## 5 The N3 and N1 class K-turns

**Fig. 7:**
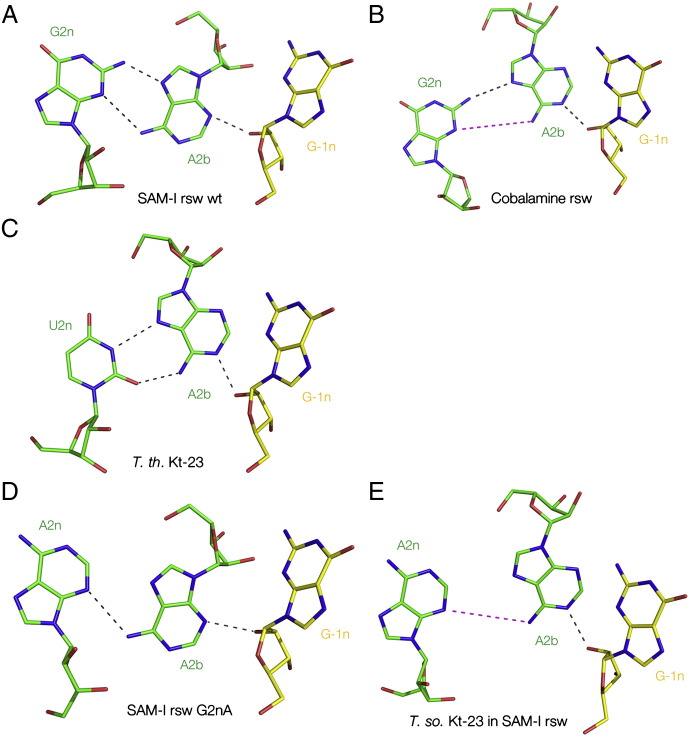
Hydrogen bonding between the 2b·2n pair and the O2′ of − 1n in N3 and N1-class K-turn structures. Hydrogen bonds are indicated by gray broken lines. Distances too long to be considered hydrogen bonded are colored magenta. A. The SAM-I riboswitch K-turn [4], a standard N3-class K-turn. B. The cobalamine riboswitch K-turn [7], an N1-class K-turn. Note that the reorientation of the A2b nucleobase results in a A2b N6–G2n N3 N–N distance that is too long for stable hydrogen bond formation. C. The *T. thermophilus* Kt-23 K-turn [36]. This K-turn has a uridine at the 2n position, making a standard Hoogsteen basepair with A2b. The latter accepts a proton from G − 1n O2′ at the N1 position, making this an N1-class K-turn. D. The SAM-I riboswitch k-turn with a G2nA substitution, making an A·A pair at the 2b·2n position. This forms a stable K-turn structure in the context of the riboswitch, for which the crystal structure has been determined [38]. A2b accepts a proton from G − 1n O2′ at the N3 position, and there is a hydrogen bond between A2b N6 and A2n N3. This is a standard N3 structure. E. The *T. solenopsae* Kt-23 K-turn, engineered into the SAM-I riboswitch. This rare K-turn sequence naturally has an adenine at the 2n position. But in the context of the riboswitch, this K-turn adopts the N1 structure [26]. A2b accepts a proton from G − 1n O2′ at the N1 position, and there is no hydrogen bond between A2b N6 and A2n N3.

The known K-turn structures are collected together in Table 1. It can be seen that these divide approximately equally between the N3 and N1 classes, in which the proton donated by the − 1n O2′ is accepted by A2b N3 or N1 respectively (Fig. 7A, B). The N3 class includes the K-turns of the SAM-I [4] and cyclic-diGMP [5] riboswitches, box C/D snoRNA [14], U4 snRNA [2] and *H. marismortui* Kt-46 [35]. In two members of this class (box C/D and U4) − 1n O2′ is also hydrogen bonded to A2b O2′. The N1 class K-turns include the cobalamine riboswitch [7] and *H. marismortui* Kt-7 and Kt-38 [35]. In three members of the N1 class (*H. marismortui* Kt-7, *T. thermophilus* Kt-23 [36] and L30e-mRNA [37]) the O2′ atoms of the − 2n and 3b ribose rings are hydrogen bonded, but this interaction is not universal in all the N1 class K-turns.

**Table 1:** Simple K-turns classified by the A2b receptor for the hydrogen bond donated by − 1n O2′; this can be N3 or N1. Inter-atomic distances (Å) for this cross-strand interaction and for the 2b·2n basepairing. Abbreviations: rsw = riboswitch, 50S = large ribosomal subunit, strain names *H. m.* = *H. marismortui*, *E. c.* = *E. coli*, *T. t.* = *T. thermophilus* and *T. s.* = *T. solenopsae*.

K-turn	PDB	− 1n: 2b/Å		2b·2n pair/Å		
		O–N3	O–N1	N2–N7	N3–N6	2b·2n
*N3 class*						
Kt-7 *H. m.* (free RNA)	4C4O	2.5		2.7	3.0	A·G
Box C/D	1RLG	2.8		2.9	3.1	A·G
U4 snRNA	1E7K	3.2		2.7	3.1	A·G
Cyclic-diGMP rsw	3Q3Z	2.8		2.7	3.0	A·G
SAM rsw	3GX5	2.6		2.5	3.4	A·G
SAM + YbxF	3V7E	3.1		3.0	3.3	A·G
T-box	4LCK	2.7		2.9	3.2	A·G
SAM rsw G2nA	2YGH	2.8			3.3	A·A

*N1 class*						
Kt-38 *H. m.*	1FFK		2.8	2.9	4.7	A·G
Kt-7 *H. m.* (50S)	1FFK		2.7	3.0	4.3	A·G
L30e	1T0K		2.7	2.7	4.6	A·G
Cobalamine rsw	4GXY		2.5	3.1	5.3	A·G
Kt-7 *E. c.*	2AWB		2.8		3.8	A·G
Kt-23 *T. t.*	2WH1		2.6			A·U
Kt-23 *T. s.*	4AEB		2.5		5.0	A·A

In order to accept a proton at N3 or N1 the rotational setting of the A2b nucleobase must change between alternative positions, and this has a consequence for the pairing with G2n. While the GN2 to AN7 hydrogen bond is preserved in both structural classes, the AN6 to GN3 distance in the N1 class structures is generally > 4 Å, so it cannot be regarded as a stable hydrogen bond.

The N3/N1 classification can be extended to the known structures of non-standard simple K-turns. *T. thermophilus* Kt-23 of the 30S ribosomal subunit [36] has a uridine at the 2n position forming a Hoogsteen basepair with A2b, but despite the difference from the standard K-turn sequence it folds normally in Mg^2 +^ ions [25]. The nucleobase of A2b was oriented so that its N1 accepts a hydrogen bond from O2′ of G − 1n, so it can be classed as an N1 K-turn (Fig. 7C). K-turns in which the 2n position is adenine are of interest because the potential A·A pair that is formed can be isosteric with the G·A pair [32]. We solved the crystal structure of a SAM-I riboswitch containing a G2nA substitution [38]. In this structure the adenine at the 2b position accepts a hydrogen bond at N3 from the O2′ of G − 1n, and it donates a single hydrogen bond from N6 to A2n N3 (Fig. 7D). This places the K-turn in the N3 class. *Thelohania solenopsae* has an extremely rare Kt-23 sequence with an adenine at the 2n position. We solved the structure of this K-turn engineered into the SAM-I riboswitch [26]. In marked contrast to the modified SAM-I K-turn sequence above, in the *T. solenopsae* K-turn A2b accepts a hydrogen bond at N1 from G − 1n O2′, and there is no hydrogen bond made with A2n (the A2b N6–A2n N3 distance is 5 Å) (Fig. 7E). This identifies *T. solenopsae* Kt-23 as a member of the N1 class. Thus K-turns with an A·A pair at the 2b·2n position have been identified in both structural classes.

So what determines whether an N3 or N1 class structure is adopted by a given K-turn? Local sequence must be important, and we have some indications that is the case. However, environment is also a factor, because the exact same sequence can adopt N3 or N1 class K-turns in different positions. The standard, simple K-turn Kt-7 of *H. marismortui* forms an N1 class K-turn structure in the large ribosomal subunit [35]. Yet the very same sequence engineered into the SAM-I riboswitch [33], or as a simple duplex RNA bound to L7Ae or free of protein [39] adopts an N3 class structure in each case. This behavior is not unique; we have found equivalent behavior for a K-turn with an A·A pair at the 2b·2n position. We have recently solved a crystal structure of the *T. solenopsae* Kt-23 as a duplex bound by L7Ae protein, where it adopts an N3 structure [40]. This clearly contrasts with the N1 class structure for the exact same sequence located in the SAM-I riboswitch [26].

This raises the question of whether or not a K-turn might exist in a dynamic equilibrium in free solution, with interconversion between the N3 and N1 class conformations. While there are no experimental data that point to such behavior at present, molecular dynamic trajectories suggest that such transient changes in hydrogen bonding pattern might be possible [41]. It is conceivable that NMR experiments might reveal this.

Comparison of N1 and N3-class Kt-7 structures reveals a coordinated change in hydrogen bonding. In addition to the absence of the A2bN6 to G2nN3 hydrogen bond in the N1 structure, the distance between the O2′ atoms at the − 2n and 3b positions varies by more than 4 Å between Kt-7 in the two conformations, being hydrogen bonded in the N1 structure [24]. This indicates that the relative disposition of the C and NC helices is affected by the change in conformation, and thus the trajectory of the helices of the K-turn. The N3–N1 conformational change was analyzed in terms of three rotation angles, describing the relative trajectory of the C-helix with respect to the NC-helix [33]. These corresponded to the axial bend angle (α), the direction of the bend (β), and the rotation around the axis of the C helix (γ). Analyzing the known K-turn structures in this way revealed a systematic difference in the angle γ between the two classes of structure. For the N3 structures the angle was < 50°, while it was > 50° for the N1 class structures. Clearly this could have a significant effect on any tertiary interactions in which the K-turn might participate. Or conversely, such interactions might influence the conformation adopted by the K-turn. We note that the natural K-turn of the SAM-I riboswitch [4] adopts the N3 conformation, while that of the cobalamine riboswitch [7] adopts the N1 structure. Each must be adapted to the local environment of its riboswitch context.

It seems that *H. marismortui* Kt-7 is probably naturally an N3 class structure, but forced to adopt the N1 structure in the ribosomal context. The terminal loop of the helix containing Kt-7 participates in a tertiary interaction with a receptor, and the K-turn is bound to the L24 protein. Either or both of these factors could be responsible for the switch to the N1 conformation.

## 6 The folding of K-turns

Fluorescence lifetime measurements have shown that in free solution without added metal ions or binding proteins, an RNA duplex containing a potential K-turn exists in a conformational equilibrium between the folded K-turn and an extended structure resembling a normal three-nucleotide bulge [42]. In the absence of added metal ions or binding proteins the equilibrium is biased towards the extended structure [42], [43]. There are three ways in which the population of folded K-turn species can be increased.

First, some (but by no means all) K-turn-forming sequences can be folded by addition of metal ions. This may be studied by the increase in the efficiency of fluorescence resonance energy transfer (FRET) between fluorophores attached to the 5′-termini of the C and NC helices with shortening of the end-to-end distance. For *H. marismortui* Kt-7, folding occurs as an all-or-none two-state process, with [Mg^2 +^]1/2 = 80 μM, and [Na^+^]1/2 = 30 mM and a Hill coefficient typically ~ 1 [24].

**Fig. 8:**
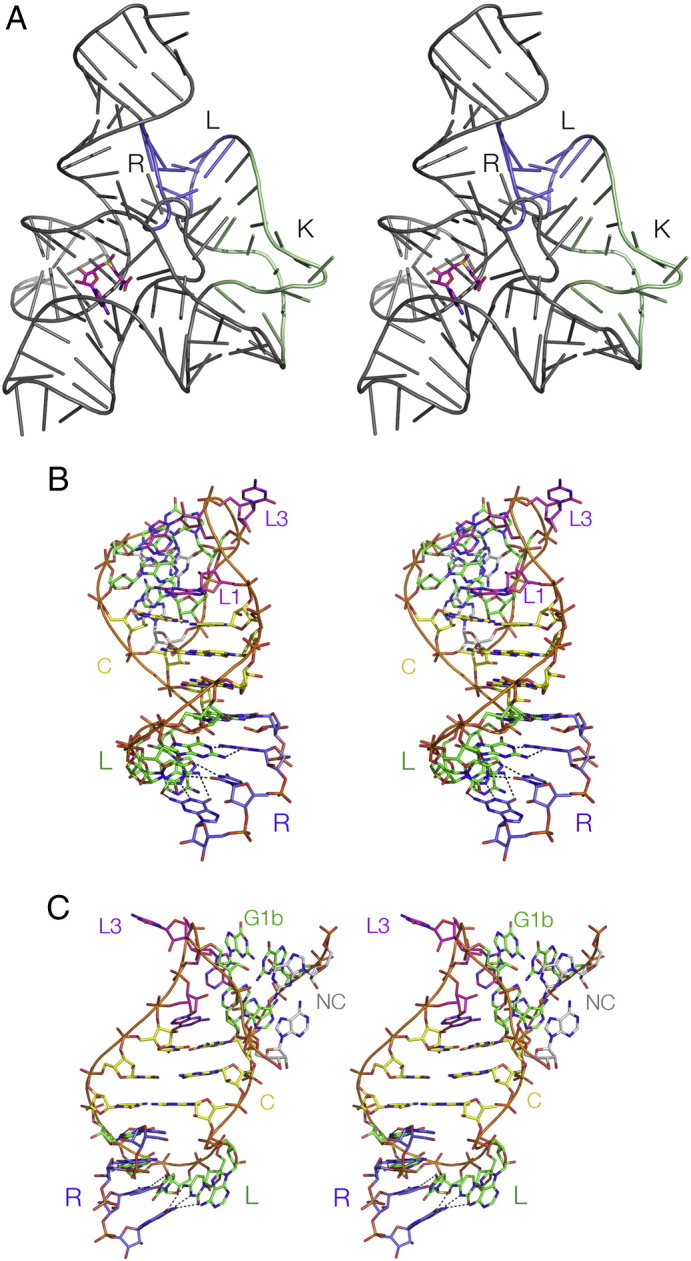
The role of the K-turn in the architecture of the SAM-I riboswitch. Molecular graphic images as parallel-eye stereoscopic views. A. Cartoon showing the structure of the complete riboswitch [4]. The K-turn (K) kinks the long helix so that its terminal loop (L) can interact with the receptor (R). This contributes to the fold of the riboswitch so that it can bind the S-adenosylmethione ligand (highlighted in magenta). B and C show two more detailed views of the K-turn and the loop–receptor interaction, with the K-turn at the top. The terminal loop of the C helix of the K-turn (colored green) is basepaired with a remote section of the riboswitch (blue).

The great majority of K-turns are involved in tertiary interactions, and this provides a second process that can stabilize the folded conformation. The change in axial trajectory at the K-turn is often used to facilitate long-range structure in large RNA molecules. Most of the ribosomal K-turns help mediate such interactions. A rather clear example can be observed in the SAM-I riboswitch, where a long helix (P2) is kinked by a standard, simple K-turn to facilitate the docking of the terminal loop into a receptor in helix P4 [4], [44] (Fig. 8). This stabilizes the global fold of the riboswitch to create a pocket in which the ligand S-adenosyl methionine (SAM) binds.

The SAM-I riboswitch system was used to demonstrate K-turn structural stabilization by the formation of tertiary interactions. In initial studies, folding was studied indirectly via the ligand binding studied by isothermal calorimetry (ITC) [38]. For example, disruption of the K-turn by an A1nC substitution led to failure to bind SAM, implying that a disruption of the 1b·1n pair prevents folding of the K-turn and consequent folding by the complete riboswitch, so that it cannot bind its ligand. But revealingly, a G2nA substitution (thus potentially creating a A·A pair at the 2b·2n position) resulted in normal binding of SAM to the riboswitch, even though the identical change in the isolated K-turn prevented ion-induced folding. We then solved the structure at 2.6 Å resolution of the modified riboswitch containing the G2nA K-turn using X-ray crystallography. This revealed that the k-turn was folded normally, and was superimposable with the unmodified K-turn with an RMSD of 0.53 Å. Thus a K-turn that cannot be folded by addition of metal ions as a simple duplex becomes folded in the context of the riboswitch, and we conclude that the overall free energy of folding of the riboswitch is coupled to that of the impaired K-turn via the tertiary interaction.

Most K-turns are binding sites for proteins, discussed in greater detail in the following section. A diverse group of proteins bind to K-turns, with varying structure and manner of interaction. The archetypal K-turn-binding proteins are the ribosomal L7Ae and related proteins. Binding of proteins provides a third process that leads to the stabilization of the folded K-turn structure.

Binding of L7Ae to *H. marismortui* Kt-7 results in folding into the kinked conformation in the absence of metal ions [45]. This can be readily demonstrated using the shortening of the end-to-end vector in a FRET experiment. But it was clear that the binding affinity is extremely high so that it was not possible to measure it in that manner. Instead it was determined indirectly as the ratio of the rates of association and dissociation measured by means of the associated conformational change. From this the apparent dissociation constant was calculated as *K*_d_ = 10 pM [31].

We have shown that other ribosomal proteins, L24 and S11, result in the folding of their cognate K-turns (L. Huang & DMJL, unpublished data). So the stabilization of K-turn structure by protein binding is quite general.

## 7 The binding of L7Ae-family proteins to K-turns

L7Ae and related proteins are a group of RNA-binding proteins that include the eukaryotic and archaeal proteins L7Ae, L30e and S12e [46], the human 15.5 kDa protein [47], and the yeast Nhp2 and Snu13p proteins, as well as bacterial homologues such as YbxF [48]. Each binds K-turn-containing RNA, and some functional substitutions are possible [49]. The formation of box C/D and H/ACA nucleoproteins that direct site-specific methylation and pseudouridylation respectively are initiated when L7Ae-type proteins bind to a K-turn located in the guide RNA [50], [51], followed by other proteins in an ordered process. A box H/ACA motif is also contained within human telomerase RNA [52]. L7Ae is a component of the U3 snoRNP [53]. The K-turn of the U4 stem-loop in the U4–U6·U5 tri-snRNP [2], [47] binds the 15.5 kDa protein. L7Ae has further been shown to be a subunit of archaeal RNaseP [54] required for tRNA maturation. The L7Ae-family proteins are rather general K-turn-binding proteins, that are important in ribosome structure, the site-specific modification of RNA, and spliceosome assembly.

**Fig. 9:**
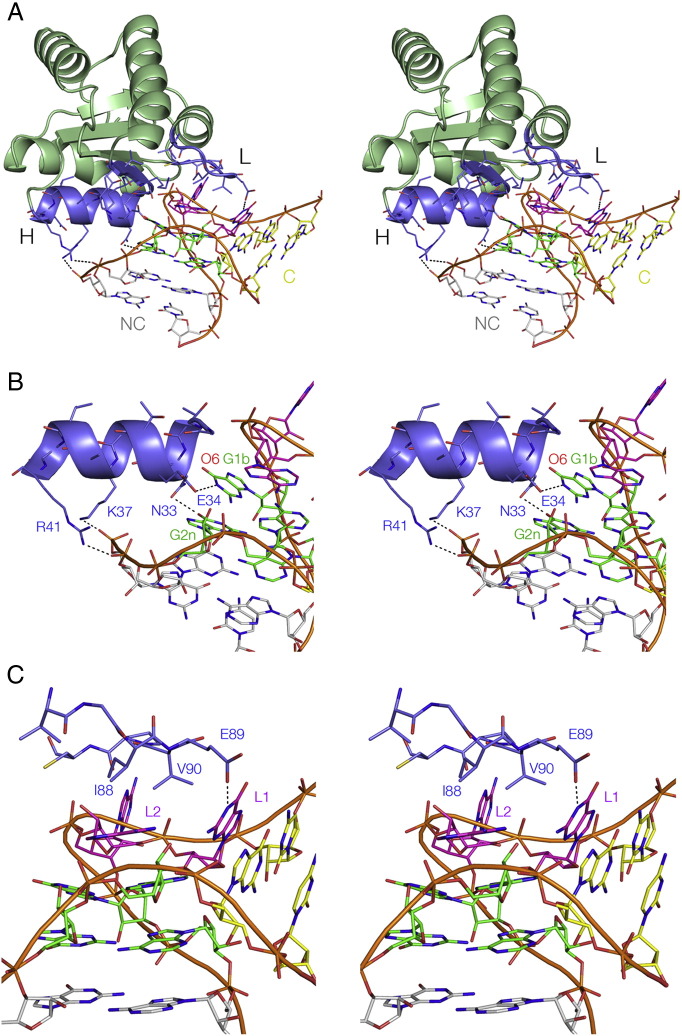
The structure of a complex between *H. marismortui* Kt-7 and *A. fulgidus* L7Ae complex, determined crystallographically at 2.3 Å resolution [39]. A. An overall view of the complex, showing the protein (depicted in cartoon form) bound in the major groove on the outer face of the K-turn. The two key regions of L7Ae involved in binding the RNA are highlighted in blue; these are the α-helix (H) and the hydrophobic loop (L). B. The α-helix interacting with the major groove of the NC helix. R41 and K37 make non-specific interactions with the backbone, while E34 and N33 make specific hydrogen bonds to the conserved guanine nucleobases G1b and G2n respectively. The electronegative O6 atom of G1b is placed at the positive pole of the helix dipole of the α-helix. C. The hydrophobic loop capping the loop region of the K-turn. The loop sits over the L2 and L1 nucleobases, with I88 and V90 on the lower face contributing to a large hydrophobic contact area. The carboxylate sidechain of E89 is hydrogen bonded to GL1 N1.

The structure of L7Ae bound to *H. marismortui* Kt-15 in the large ribosomal subunit has been available since the 50S subunit was solved [35]. In addition, crystal structures of the complexes of L7Ae with a box C/D K-turn [14], and the human 15.5 kDa protein with the U4 snRNA K-turn [2] have been determined. We recently solved the structure of *Archaeoglobus fulgidus* L7Ae bound to the well-studied *H. marismortui* Kt-7[39] (Fig. 9). The structure was solved at a resolution of 2.3 Å, the highest available for a standard K-turn complex, and comparing the structure with the other complexes allows us to establish the general principles for the recognition of K-turn structure by the L7Ae proteins.

These proteins bind in the major groove of the K-turn that runs around the outside of the structure. The major groove of normal duplex RNA is deep and narrow and so inaccessible to protein. However the structure of the K-turn splays open the groove, making it very accessible. The binding interface of L7Ae-related proteins comprises two elements. The first is a highly basic β-strand: turn: α-helix element, and the second a short loop of hydrophobic residues (plus a glutamate at one end) that makes a 180° turn.

The three-turn α-helix enters the major groove of the NC helix, directing its N-terminal end towards the major groove edges of the G1b and G2n nucleobases of the G·A pairs (Fig. 9B). This resembles the entry of the recognition helices of helix-turn-helix elements interacting with DNA. The C-terminus of the α-helix lies close to the non-bulge-containing strand of the NC RNA helix, and contributes non-specific interactions to the binding. The arginine in the third turn (R41-residue numbering here refers to *A. fulgidus* L7Ae) hydrogen bonds to the 3n/4n phosphate. This interaction is near-universal, proposed in the complexes of the same species L7Ae with box C/D and the human 15.5 kDa protein with U4 snRNA. The lysine at the second turn (K37) is more variable in its interactions, but is bonded to the 4n/5n phosphate in the Kt-7 complex. Two of the basic side chains at the N-terminal end of the β-sheet section are also directed towards the NC helix non-bulged strand. These are not close enough for hydrogen bonding, but should contribute to overall electrostatic stabilization. The N-terminal end of the α-helix is involved in specific recognition of the core of the K-turn. The glutamate side chain from the first turn (E34) is hydrogen bonded to N1 of G1b. This interaction is also observed in the interactions of L7Ae with box C/D and the 15.5 kDa protein with U4 snRNA, and is likely to be universal. The adjacent asparagine residue (N33) is hydrogen bonded to O6 of G2n, also observed in the box C/D interaction. In the L7Ae–Kt-7 complex, as well as the other structures considered here, the O6 atom of G1b is located almost on the axis of the α-helix at the N-terminal end. Guanine O6 has a significant partial negative charge, and this will be electrostatically stabilized by the positive pole of the helix dipole. This appears to be another universal interaction in the L7Ae–K-turn complexes.

The hydrophobic loop covers the nucleobases in the L2 and L1 positions (Fig. 9C), making good Van der Waals contact and burying a surface area of 732 Å^2^. The syn conformation of L2 places it maximally in contact with the loop. Isoleucine I88 in particular is located directly over GL2 in the Kt-7 complex. A very similar interaction is observed in the box C/D and 15.5-U4 snRNA complexes. The glutamate of the loop (E89) is hydrogen bonded to N1 of the L1 guanine, thus making a specific contact.

Taken together, these elements generate a very specific molecular recognition of the structure of the K-turn in double-stranded RNA. By placing the α-helix in the major groove of the NC arm and capping the L2 nucleobase it effectively ‘measures’ the angle between these two elements in the major groove. This then juxtaposes the N-terminus of the α-helix with the major groove side of the guanine bases of the two conserved G·A, where side chains make specific hydrogen bonds, and G1b O6 is placed to maximize the interaction with the positive pole of the helix dipole.

The folding of Kt-7 that occurs on the binding of L7Ae might in principle occur by a passive conformational selection process [55], or by a more active induced fit process [56]. Parenthetically it should be said that we tend to use the term ‘induced fit’ somewhat loosely referring to K-turn formation on protein binding, but now we are giving it a more specific meaning. We have studied the binding of Kt-7 to L7Ae in single molecule experiments. In studying the binding of single Kt-7 RNA molecules to L7Ae in real time we could not detect any intermediate folded state down to a timescale of 8 ms [57]. This is consistent with a conformational selection process, although we cannot exclude that a protein-induced conformational change occurs in the RNA on a faster timescale. There are two requirements for a conformational selection process to be possible. First, the folded form must be in equilibrium with the unfolded form prior to binding, and we have shown that this is the case in time-resolved FRET experiments [42]. Second, the structure of the unbound but folded RNA structure must be closely similar to that of the protein-bound RNA. Comparison of our two crystal structures of Kt-7 free and bound by L7Ae shows that the structures are indeed closely similar [39], with an all-atom RMSD of 0.83 Å. So no conformational adjustment of the RNA structure should be required on binding to L7Ae. Thus all the prerequisites for the conformational selection process to occur exist, and this is by far the most likely process by which the folding of the population occurs.

## 8 The roles of K-turns in the folding and architecture of RNA

The K-turn motif generates an abrupt change in the trajectory of the axis of duplex RNA. In many cases either the C or NC helix has a terminal loop that is involved in a tertiary interaction, and this is clearly important in facilitating the long-range architecture of RNA molecules. This is true both in relatively small RNA species such as many of the riboswitches and also in large structures like the ribosome. We have seen that in the SAM-I riboswitch the C-helix of the K-turn is involved in a loop–receptor interaction that stabilizes the fold of the functional riboswitch [4]. A very similar interaction is likely to occur in the *B. subtilis* lysine riboswitch where the terminal loop of the C-helix probably makes a loop–loop interaction [9]. In the cyclic-diGMP riboswitch the strands of the C-helix of the K-turn splays apart to create a part of the ligand binding site; the other part comprises an extensive loop–loop interaction that is mediated by the NC helix of the K-turn [5], [58]. A rather long helix of the cobalamine riboswitch is kinked by its K-turn [7], while the K-turn of the glycine riboswitch connects the two aptamer domains mediating their association that leads to cooperative ligand binding [10].

Many of the K-turns also function as the binding sites for specific proteins. The folded, tightly-kinked structure of the K-turn can be stabilized by addition of metal ions for some, but not all sequences, by the binding of specific proteins including the L7Ae family, and by tertiary interactions within a larger RNA structure. There are numerous cases in which the biogenesis of ribonuclear species use one or more of these processes. The first event in the formation of the box C/D snoRNA occurs when an L7Ae-family protein binds to a K-turn [50], [51]. This then provides a platform on which to recruit further proteins, culminating with the methyl transferase enzyme that carries out the site-specific modification of the RNA.

The majority of ribosomal K-turns mediate short- or longer-range tertiary interactions, and bind specific proteins, so we would expect that interplay between tertiary interactions and protein binding would be very important to the folding path. It could be envisaged that the K-turns are initially relatively flexible in an exchange between folded and unfolded conformations, allowing the local RNA to explore tertiary contacts. Flipping between N3 and N1 conformations might also be important during this process. As the contacts are found the structure could then become fixed by the binding of proteins. An example of this is provided by the 5′-terminal region of the 23S rRNA of *H. marismortui* [35]. Kt-7 is located in one arm of a three-way junction. The terminal loop of the C helix interacts with a receptor in the adjacent helical arm. The kink generated by the K-turn is an essential structural requirement for this. Kt-7 is bound by L24, which binds early in the 50S subunit assembly according to Nierhaus [59], [60], and we have shown that L24 binding induces Kt-7 to adopt the kinked conformation (L. Huang and DMJL, unpublished data). Thus this section of the ribosome is likely to fold in response to both tertiary contacts (the three-way helical junction and the loop–receptor interaction) and protein binding (L24). Perhaps this provides a simple model for the folding of the complete ribosomal subunit.
